# The Role of Global and Local Visual Information during Gaze-Cued Orienting of Attention

**DOI:** 10.1371/journal.pone.0160405

**Published:** 2016-08-25

**Authors:** Nicolette M. Munsters, Carlijn van den Boomen, Ignace T. C. Hooge, Chantal Kemner

**Affiliations:** 1 Department of Experimental Psychology, Helmholtz Institute, Utrecht University, Utrecht, The Netherlands; 2 Department of Developmental Psychology, Utrecht University, Utrecht, The Netherlands; 3 Department of Child and Adolescent Psychiatry, Brain Center Rudolf Magnus, University Medical Center, Utrecht, The Netherlands; Harvard Medical School, UNITED STATES

## Abstract

Gaze direction is an important social communication tool. Global and local visual information are known to play specific roles in processing socially relevant information from a face. The current study investigated whether global visual information has a primary role during gaze-cued orienting of attention and, as such, may influence quality of interaction. Adults performed a gaze-cueing task in which a centrally presented face cued (valid or invalid) the location of a peripheral target through a gaze shift. We measured brain activity (electroencephalography) towards the cue and target and behavioral responses (manual and saccadic reaction times) towards the target. The faces contained global (i.e. lower spatial frequencies), local (i.e. higher spatial frequencies), or a selection of both global and local (i.e. mid-band spatial frequencies) visual information. We found a gaze cue-validity effect (i.e. valid versus invalid), but no interaction effects with spatial frequency content. Furthermore, behavioral responses towards the target were in all cue conditions slower when lower spatial frequencies were not present in the gaze cue. These results suggest that whereas gaze-cued orienting of attention can be driven by both global and local visual information, global visual information determines the speed of behavioral responses towards other entities appearing in the surrounding of gaze cue stimuli.

## 1. Introduction

The use of eye gaze has, besides mere visual detection, several social-cognitive functions (e.g. [1,2]). In social situations eye gaze is an important social communication tool, and is referred to as social gaze. Any gaze-based interaction starts with eye contact between two individuals, a situation designated as mutual gaze [2]. Mutual gaze indicates communicative intent and ‘opens the channel’ of social interaction [2,3]. Subsequent averted gaze may provide a cue that another individual’s attention has shifted toward another entity (e.g. person, event, object). Averted gaze often results in a reflexive corresponding shift of attention in the observer (defined as gaze following; e.g. [4]). Gaze direction frequently provides crucial information about the attention and potentially the intentions of people. Therefore, gaze following is considered essential for the understanding of others [2]. Since timing of responses is an important aspect of the quality of social interaction, increased insight in factors that affect the speed of gaze-cued orienting of attention could shed light on determinants of successful social interaction. Previous research already showed that global information, representing the configuration of the face, and more detailed local visual information, such as sharp edges, play specific roles in processing other socially relevant information from a face (e.g. [5]). However, studies that investigated the role of global and local visual information during gaze-cued orienting of attention show mixed results (e.g. [6–10]). The present study aimed to more thoroughly investigate the role of global and local visual information during gaze-cued orienting of attention in adults, by using multiple measures and well-described stimuli.

Theoretical models predicted that global visual information has a primary role during social gaze processing [11,12]. For example, a gaze shift from one individual results in gaze following in another individual when global visual information is present, but this is attenuated when only local visual information is available. This was mainly based on the suggestion that brain areas involved in social gaze processing receive primarily information from the fast magnocellular subcortical and dorsal pathway tuned to global visual information, instead of the slower parvocellular ventral pathway tuned to local visual information [13–16]. Indeed, previous results showed that gaze-cued orienting of attention was primarily driven by global visual information [6–8,10]. In contradiction, other results showed that gaze-cued orienting of attention was driven by both global and local visual information (e.g. [6,7,9,10]). These latter results oppose a primary role of global visual information during gaze-cued orienting of attention. In sum, previous research shows conflicting findings about the role of global and local visual information during gaze-cued orienting of attention.

An explanatory factor for the mixed findings in previous research might be the stimuli that are used to investigate responses during gaze-cued orienting of attention. We suggest that the operationalization of global and local visual information by spatial frequencies [6] results in a better description of the visual content of the stimuli than adjusting the visual content by creating blurred and line-drawn faces [7] or upright and inverted faces (e.g. [8–10]). Even though line-drawn faces and un-manipulated faces appear very different both contain broadband visual information. Similarly, even though upright faces and inverted faces appear very different both contain local visual information. In contrast, by filtering the local and global faces to contain specific spatial frequency content the faces can be clearly discriminated on visual content from faces without manipulation. These well-described stimuli allow investigating whether specific spatial frequency bands, related to global and local visual information, play a role during gaze-cued orienting of attention.

Another factor that might influence research results is the measure that is used to study gaze-cued orienting of attention. Previous research applied varying measures for this investigation. The manual responses, investigated in the behavioral research, might be less sensitive for differences in timing of gaze-cued orienting of attention than the neural responses measured with electroencephalography (EEG), explaining the lack of differences between the local and global condition in some of the behavioral studies. Furthermore, since a social gaze based interaction involves the eye movements of both the gazer and the follower [2], eye movements (i.e. saccadic responses) are probably a more relevant behavioral aspect of social gaze than manual responses. Thus, saccadic and neural responses might be more sensitive and relevant measures to detect differences in gaze-cued orienting of attention when local or global visual information is present, than manual responses. Overall, the different measures are complementary and together provide insight in the timing and sequence of gaze-cued orienting of attention: from neural processing of a gaze cue until saccades in response to subsequent entities in the proximity of the gaze cue.

The aim of the present study is to investigate in adults, with multiple measures (i.e. manual responses, saccadic responses and neural responses) and well-described stimuli (i.e. manipulated for spatial frequency content), whether gaze-cued orienting of attention is primarily driven by global visual information and diminished when only local visual information is available. Studies typically apply a gaze-cueing task in which a centrally presented face cued validly or invalidly the location of a peripheral target through a gaze shift. Participants need to indicate with a key press as fast as possible after target onset where the target appeared. Behavioral responses towards the target are usually faster for correctly cued targets than incorrectly cued targets [17,18]. This is known as a cue-validity effect, which probably reflects a reflexive covert shift of attention elicited by the gaze cue (i.e. covert gaze following; [18]). Furthermore, in the present study also saccadic reaction times (i.e. reaction times of eye movements) towards the target are investigated, as these are probably a more important behavioral aspect of social gaze. These overt shifts of attention towards the target are probably influenced by covert shifts of attention elicited by the gaze cue resulting in a cue-validity effect. Additionally, as in previous research, we measured neural responses (i.e. electroencephalography (EEG)), since this method has a high temporal resolution and is therefore very sensitive for differences in processing time. Neural responses towards the target, reflected in the occipitotemporal P1 and N200 peak in the event-related potential (ERP), are typically earlier and larger for correctly cued targets than incorrectly cued targets. This is assumed to reflect increased neural activity in the extrastriatal visual cortex to facilitate the processing of attended stimuli [19,20]. Furthermore, during gaze-cue presentation, attention orienting and holding to the cued side can be investigated through the laterality effect (i.e. more negative and less positive ERP amplitude for faces with gaze directed to the contralateral side compared to faces with gaze directed to the ipsilateral side) on the posterior early directing attention negativity (EDAN) and the anterior directing attention negativity (ADAN) [21,22]. The EDAN and ADAN can provide insight in the neural mechanisms leading to faster responses to the validly cued target. We expected gaze-cued orienting of attention, visible in 1) a gaze laterality effect on ADAN and EDAN during cue presentation, and 2) shorter manual and saccadic reaction times and an earlier and larger P1 and N200 peak for the correctly cued targets compared to the incorrectly cued targets (i.e. cue-validity effect) during target-presentation.

Furthermore, in the present study the presented faces contain the lower (LSF), the mid-band (MSF), the higher (HSF), or all (unfiltered; UF) spatial frequency information present in the original picture of the face. LSF (large-scale luminance variations) are suggested to support, more than HSF (small-scale luminance variations), the processing of the global configuration of the face (i.e. global visual information). In contrast, HSF are suggested to accentuate, more than LSF, the encoding of detailed visual information such as sharp edges present in a face (i.e. local visual information; [23,24]). In the literature often a third category is distinguished, containing the mid-band spatial frequencies (MSF): the visual information in between LSF and HSF [25]. MSF is added as an extra condition, because MSF is known to be important for face perception [26]. We expected 1) the gaze laterality effect (EDAN, ADAN) to interact with spatial frequency and 2) an interaction of cue-validity and spatial frequency on responses towards the target for saccadic reaction times and neural responses and not for manual reaction times. Specifically, gaze laterality effects (EDAN, ADAN) and cue-validity effects (at P1, N200 and in saccadic reaction times) in the LSF condition (containing global visual information) and MSF conditions (containing some global and local visual information) are expected to be comparable to effects in an unfiltered condition. Attenuated gaze laterality effects (EDAN, ADAN) and cue-validity effects (at P1, N200 and in saccadic reaction times) are expected in the HSF condition (containing only local visual information). These effects would suggest that the gaze-cued orienting of attention is primarily driven by global visual information and diminished when only local visual information is available.

## 2. Material and Methods

### 2.1 Participants

In total 47 participants between 18 and 30 year old were recruited through posters and flyers at the Utrecht University (The Netherlands). Participants were excluded from data analysis if their vision was abnormal and not corrected-to-normal based on self-report (N = 3), or due to insufficient data quality (eye tracking N = 6; the criteria for sufficient data quality are described in the data analysis section) or technical errors (EEG and reaction times N = 2; eye tracking N = 2). The final analyses were done on data of 42 participants (16 male; 21.4 mean age) for the EEG and reaction time task and 36 participants (13 male; 21.4 mean age) for the eye tracking task. The Ethics Committee of the Utrecht University Social Science Faculty approved this study. Participants gave written informed consent prior to participation.

### 2.2 Stimuli

**Fig 1:**
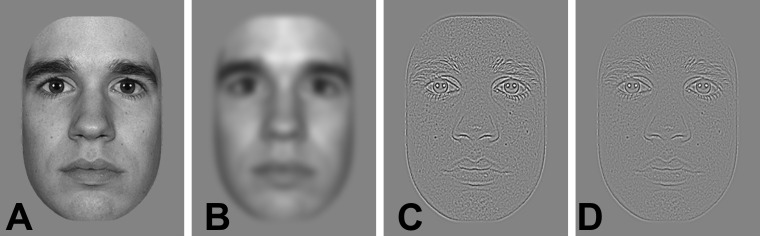
**Example of an unfiltered face stimulus (A) and its low-pass (B), mid-pass (C) and high-pass (D) filtered versions.** The face in this figure is derived from the MacBrain Face Stimulus Set, the model approved publication in scientific journals.

Face stimuli consisted of 10 photographs of faces with neutral facial expression taken from the MacBrain Face Stimulus Set (Development of the MacBrain Face Stimulus Set was overseen by Nim Tottenham and supported by the John D. and Catherine T. MacArthur Foundation Research Network on Early Experience and Brain Development. Please contact Nim Tottenham at tott0006@tc.umn.edu for information concerning the stimulus set). Face images included 5 males and 5 females, of which 6 European-American, 3 African-American and 1 Asian-American model. Face pictures were trimmed to remove external features (neck, ears, and hairline). Using Photoshop straight and averted gaze were created, and all faces were cropped, turned into gray-scale and matched for size (560 by 820 pixels; 12.9° by 18.5° of visual angle at a viewing distance of 65 cm). Four conditions were created: the faces were either unfiltered (broadband; UF) or filtered with a low- (LSF; <2 cycles per degree), mid- (MSF; 2–6 cycles per degree) or high-pass (HSF; >6 cycles per degree) spatial frequency filter (see Fig 1). These cutoffs were taken from previous literature (e.g. [5]). Filtering was performed in Matlab (The Mathworks, Natick, MA) using a set of Gaussian filters. The LSF, MSF and HSF stimuli still differed in terms of global Root Mean Square (RMS) contrast from broadband (UF: 33 cd/m^2^; LSF: 36 cd/m^2^; MSF: 13 cd/m^2^; HSF: 14 cd/m^2^). RMS contrast has been shown to be the best index for perceived contrast in natural images [27].

### 2.3 Procedure

Participants were seated in a quiet and dimly room and rested their chin and forehead on a desk-mounted headrest positioned at eye level 65 cm from the computer screen, while wearing the EEG cap. The participants performed two tasks in counterbalanced order, in which either EEG together with manual reaction times were measured (i.e. EEG-task) or saccadic reaction times with eye tracking (i.e. ET-task). The EEG and eye-tracking experiments were not performed simultaneously as this would lead to eye artifacts in the EEG data. Both tasks consisted of a 4 (SF: UF, LSF, MSF, HSF) x 2 (validity: invalid, valid) conditions design. Stimuli were the same across tasks and presented in two blocks, in random order of 40 (EEG-task) or 20 (ET-task) stimuli per condition, on a 23-inch screen with a resolution of 1920x1080 pixels, and a refresh rate of 60 Hz.

**Fig 2:**
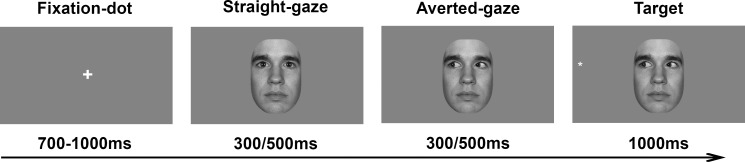
The stimuli and timing sequence used in the EEG (500ms) and ET (300ms) task. The illustration shows a valid trial. The face in this figure is derived from the MacBrain Face Stimulus Set, the model approved publication in scientific journals.

Each trial started with a fixation-dot presented in the middle of the screen (jittered duration between 700-1000ms; see Fig 2). Next, a face with straight gaze was presented, followed by the same face with averted gaze to randomly the left or the right. Then a target cross (subtending 1.2°) was placed counterbalanced on the left or right side of the screen for 1000ms (19.6° off center) and was either valid or invalid with the gaze-cue. During target presentation, the averted-gaze remained visible on the screen to avoid offset effects in the EEG. Moreover, in the EEG-task the time between the straight-gaze onset and the averted-gaze onset, and the time between the averted-gaze onset and target onset were both 500ms, since shorter stimulus-onset asynchrony would lead to overlap between EEG responses reflecting the visual processing of the cue and the peaks of interest in response to the target. However, de Jong (2008) suggested a possible lack of differences in processing speed for low and high spatial frequencies in their study could be due to the long cue duration in their study. Therefore, and to create a more ecological valid design, in the ET-task the time between the straight-gaze onset and the averted-gaze onset, and the time between the averted-gaze onset and target onset were both 300ms. Stimuli were presented on a gray background matching the average luminance of the face stimuli (see Fig 2).

Participants were instructed that gaze direction did not predict target location. Furthermore, in the EEG-task participants were instructed to fixate on the central face throughout the experiment and to respond to appearance of the target by pressing a corresponding left or right button as quickly and accurately as possible. In the ET-task participants were instructed to fixate on the target as quickly and accurately as possible.

### 2.4 Data Analyses

#### 2.4.1 EEG

EEG data was recorded with 32 electrodes (Active Two system, Biosemi) positioned at standard recording locations in a cap according to the international 10/20 system. Two extra electrodes, the CMS (Common Mode Sense) and DRL (Driven Right Leg), provided an active ground. In addition, horizontal and vertical electro-oculograms were measured, with electrodes placed above, under and next to the left eye and one next to the right eye. EEG was sampled at 2048 Hz and re-sampled offline with spline interpolation to 512 Hz. Using Brain Vision Analyser software (Brainproducts GmbH) data was filtered with a high-pass filter of 0.1 Hz (24 dB/oct), a low-pass filter of 30 Hz (24 dB/oct) and a notch filter of 50 Hz. Epochs from 100ms pre-gaze-cue-onset to gaze-cue-onset for the gaze cue ERP’s (i.e. ADAN, EDAN) and 100ms pre-straight-gaze until target-offset for the target ERP’s (i.e. P1, N200) were extracted from the continuous data. Eye movement artifacts (using the algorithm by [28]) were corrected and EEG artifacts (defined as amplitudes >±100μV, amplitude differences within 200 milliseconds <3μV, voltage steps per sample point >50μV) were removed. Next a baseline correction was performed, with baseline defined as 100ms pre-gaze-cue-onset to gaze-cue-onset for the gaze cue ERP’s and 100ms pre-target-onset to target-onset for the target ERP’s. All of the electrodes were referenced to the average of all electrodes. Electrodes that contained less than 30 segments per condition were defined as bad measurement and not included in the reference and average ERP. For each stimulus condition an average ERP was created from 100ms pre-gaze-cue-onset to gaze-cue-onset for the gaze cue ERP’s and 100ms pre-target-onset to target-offset for the target ERP’s. All included participants had at least 30 included segments in the ERP at the electrodes of interest. One participant was excluded from the gaze-cue ERP analyses since there were less then 30 segments at the electrode of interest.

The peaks of interest were the EDAN and ADAN during gaze-cue presentation and the P1 and N200 during target presentation. Based on previous studies (e.g. [10]) and after data inspection the EDAN was measured at P7 and P8 between 200 and 300ms after gaze-cue-onset, whereas the ADAN was measured at F7, F8, FC5 and FC6 between 300 and 500ms after gaze-cue-onset. For each component, the mean amplitude across the defined time window (corrected for amplitude at gaze-cue-onset by subtracting gaze-cue-onset amplitude from mean amplitude) was averaged across the electrodes for a given hemisphere and exported for further analyses. More negative and less positive amplitude for faces with gaze directed to the contralateral side compared to faces with gaze directed to the ipsilateral side (i.e. gaze laterality effect) is indicative of EDAN and ADAN respectively. The P1 and N200 were, based on previous results [6] and data inspection, automatically scored and manually checked at P3 between 90 and 160ms after target-onset for P1 and between 150 and 220ms after target onset for N200. For each participant peak amplitude (corrected for amplitude at target-onset by subtracting target-onset amplitude from peak amplitude) and latency of the P1 and N200 per condition were exported for further analyses.

#### 2.4.2 Manual reaction times

Manual reaction times were measured during the EEG-task. Trials were included in analyses if they included a response corresponding to the target position (i.e. correct response) and the response was given at least 80ms after target-onset (i.e. no anticipatory responses were included). Trials with extreme reaction times (>3 SD from the mean; 0.5% of the data) were excluded from analyses. Participants gave a response corresponding to the target position on 98% of the trials, on average 97% of the trials were included in analyses (i.e. no trials with anticipatory responses were included). For each participant the median of manual reaction times per condition were calculated for further analyses. Manual reaction times were defined as the time between target onset and the time point at which the participant gave a response corresponding to the target position.

#### 2.4.3 Eye tracking

Eye movement data were recorded with the Eyetech TM3 at 52Hz. Fixations were detected using a program that marked fixations by an adaptive velocity threshold method [29]. Two participants were excluded from analysis as their average precision (RMS; root mean square; [30]) was above 6° degrees (compared to an average precision of 0.45° ± 0.36° of the included participants). Trials were included in analyses if they included a fixation during target presentation on or around target position and the preceding fixations were on the face-stimuli till at least 80ms after target-onset (i.e. no anticipatory saccades were included). Four participants were excluded from analyses, because there were no included trials in one or more conditions due to anticipatory saccades in most of the trials. Trials with extreme reaction times (>3 SD from the mean; 1.0% of the data) were excluded from analyses. The included participants fixated on the target in 99% of the trials, on average 76% of the trials (at least 5 trials per condition) were included in analyses (i.e. no trials with anticipatory saccades were included). For each participant median saccade latencies of target fixations per condition were calculated for further analyses. Saccadic reaction time of target fixation was defined as the time between target onset and moment of saccade start towards the target.

### 2.5 Statistical Analyses

Repeated measures analyses of variance were performed for neural responses during presentation of the gaze-cue with filtering (UF, LSF, MSF, HSF), gaze laterality (contralateral, ipsilateral) and hemisphere (left, right) as independent variables and either EDAN or ADAN amplitude as dependent variable. Furthermore repeated measures analyses of variance were performed for responses during the target with filtering (UF, LSF, MSF, HSF) and cue-validity (invalid, valid) as independent variables and either N200 amplitude, N200 latency, manual reaction time, or saccadic reaction time as dependent variable. When the overall effect of filtering or the interaction between filtering and ADAN, EDAN or cue-validity was significant, simple contrast were performed to compare the filtered conditions to the broadband unfiltered condition. For all reported analyses, the alpha value was set at .05 and all contrasts are corrected for multiple testing using Bonferroni correction.

## 3. Results

### 3.1 Responses during Gaze-Cue Presentation

#### 3.1.1 EDAN

**Fig 3:**
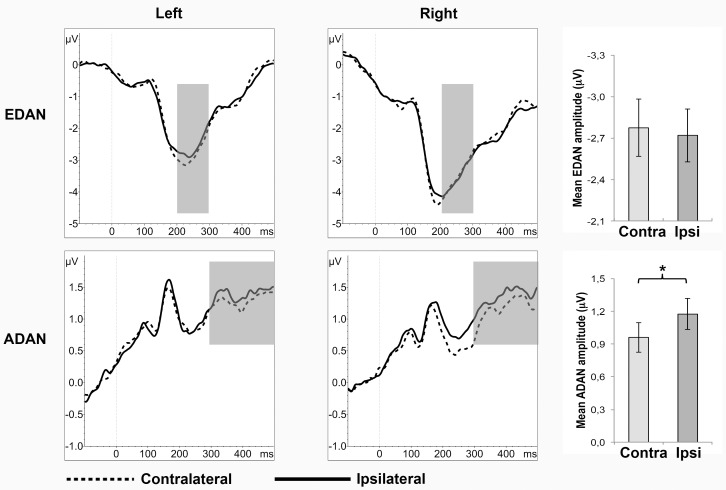
ERP waveforms and mean amplitude ± SE of the EDAN and ADAN for contralateral (contra) and ipsilateral (ipsi) gaze cues. *P < 0.05.

There was no significant main effect of gaze laterality (F_(1,40)_ = 0.25; P = 0.622; η^2^ = 0.01; see Fig 3), nor an interaction effect of gaze laterality * filter (F_(1,40)_ = 0.04; P = 0.990; η^2^ < 0.01). There was a significant main effect of hemisphere (F_(1,40)_ = 4.27; P = 0.045; η^2^ = 0.10). Amplitudes were more negative in the right than the left hemisphere. No other significant effects were found (all P > .23; all η^2^ < .04).

#### 3.1.2 ADAN

There was a significant main effect of gaze laterality (F_(1,40)_ = 6.40; P = 0.015; η^2^ = 0.14), amplitudes were less positive for contralateral than ipsilateral gaze (see Fig 3), but not an interaction effect of gaze laterality * filter (F_(1,40)_ = 0.43; P = 0.733; η^2^ = 0.01). Furthermore, there was an interaction effect of filter * hemisphere (F_(3,120)_ = 3.82; P = 0.012; η^2^ = 0.09). Contrasts revealed that the ADAN for MSF was larger on the right than left hemisphere and this difference was significantly stronger for MSF than UF (UF<MSF F_(1,40)_ = 6.43, P = 0.045, η^2^ = 0.14). The LSF and HSF condition did not differ from UF (UF-LSF F_(1,40)_ = 2.30, P = 0.411, η^2^ = 0.05; UF-HSF F_(1,40)_ = 0.01, P = 1.000, η^2^ = 0.00). No other significant effects were found (all P > .39; all η^2^ < .02).

### 3.2 Responses during Target Presentation

#### 3.2.1 Cue-validity * Filtering

Mauchly’s test indicated that the assumption of sphericity had been violated for the interaction effect of filter *cue-validity for saccadic reaction time (χ^2^_(5)_ = 18.50, P = .002). Therefore degrees of freedom were corrected using Greenhouse-Geisser estimates of sphericity (ε = .81 for saccadic reaction time). There was no significant interaction effect between filtering and cue-validity for P1 amplitude (F_(3, 123)_ = 0.78, P = 0.508; η^2^ = 0.02), P1 latency (F_(3, 123)_ = 0.62, P = 0.605; η^2^ = 0.02), N200 amplitude (F_(3, 123)_ = 0.39, P = 0.762; η^2^ = 0.01), N200 latency (F_(3, 123)_ = 0.26, P = 0.857; η^2^ = 0.01), manual reaction times (F_(3, 123)_ = 2.13, P = 0.100; η^2^ = 0.05) or saccadic reaction time (F_(2.4, 84.9)_ = 0.69, P = 0.530; η^2^ = 0.02).

#### 3.2.2 Cue-validity

**Fig 4:**
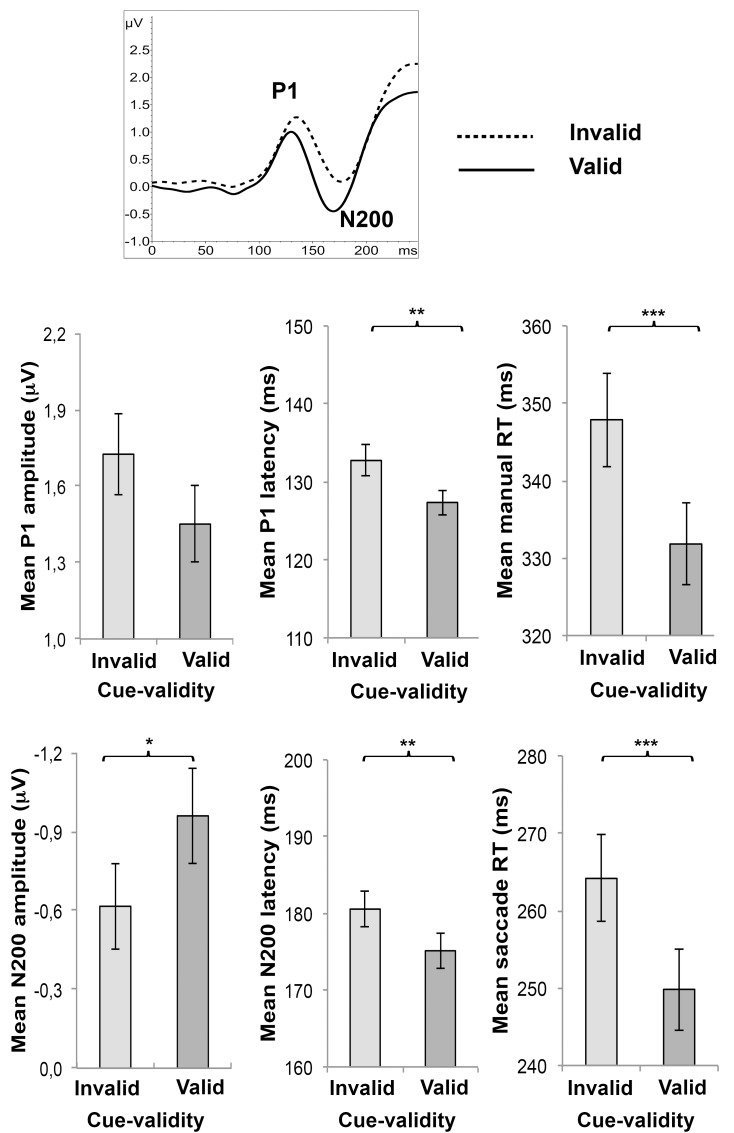
ERP waveforms and mean amplitude ± SE of P1 amplitude, P1 latency, N200 amplitude, N200 latency, manual reaction times (RT) and saccadic reaction times (RT) for each cue-validity condition. *P < 0.05; **P < 0.01; ***P < 0.001.

There was a significant main effect of cue-validity for P1 latency (F_(1,41)_ = 11.05; P = 0.002; η^2^ = 0.21), N200 amplitude (F_(1,41)_ = 4.37; P = 0.043; η^2^ = 0.10), N200 latency (F_(1,41)_ = 10.20; P = 0.003; η^2^ = 0.20), manual reaction times (F_(1,41)_ = 60.71; P < 0.001; η^2^ = 0.60) and saccadic reaction times (F_(1,35)_ = 40.29; P < 0.001; η^2^ = 0.54). Invalid targets elicited smaller and later N200 peaks, longer manual and saccadic reaction times than valid targets (see Fig 4). No significant main effect of cue-validity was found for P1 amplitude (F_(1,41)_ = 3.84; P = 0.057; η^2^ = 0.09).

#### 3.2.3 Filtering

**Fig 5:**
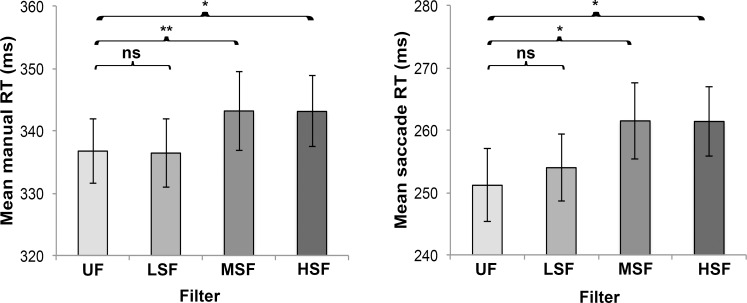
Mean ± SE of manual reaction times (RT) and saccadic reaction times (RT) for each filter condition. *P < 0.05; **P < 0.01; ns = not significant.

Mauchly’s test indicated that the assumption of sphericity had been violated for the main effect of filtering for manual reaction times (χ^2^_(5)_ = 13.77, P = 0.017). Therefore degrees of freedom were corrected using Greenhouse-Geisser estimates of sphericity (ε = .85 for manual reaction times). There was no significant main effect of filtering for P1 amplitude (F_(3, 123)_ = 1.67; P = 0.177; η^2^ = 0.04), P1 latency (F_(3, 123)_ = 1.41; P = 0.243; η^2^ = 0.03), N200 amplitude (F_(3, 123)_ = 0.51; P = 0.678; η^2^ = 0.01) and N200 latency (F_(3, 123)_ = 0.28; P = 0.840; η^2^ = 0.01). There was a significant main effect of filtering for manual reaction times (F_(2.5, 104.4)_ = 8.00, P < 0.001; η^2^ = 0.16) and saccadic reaction times (F_(3, 105)_ = 4.84, P = 0.003; η^2^ = 0.12). Contrasts revealed that manual reaction times (UF<HSF F_(1,41)_ = 9.41, P = 0.012, η^2^ = 0.19; UF<MSF F_(1,41)_ = 10.73, P = 0.006, η^2^ = 0.21) and saccadic reaction times (UF<HSF F_(1,35)_ = 8.89, P = 0.015, η^2^ = 0.20; UF<MSF F_(1,35)_ = 8.62, P = 0.018, η^2^ = 0.20) were significantly longer for HSF than UF and MSF than UF (see Fig 5). No differences were revealed between UF and LSF (manual reaction times: F_(1,41)_ = 4.23, P = 1.00; saccadic reaction times: F_(1,35)_ = 0.79, P = 1.00).

## 4. Discussion

The aim of the present study was to investigate in adults, with neural and behavioral measures and well-described stimuli (i.e. filtered for spatial frequency content), whether global visual information, as opposed to local visual information, has a primary role during gaze-cued orienting of attention. We presented a localization task in which a centrally presented face (filtered to contain specific visual information) cued validly or invalidly the location of a peripheral target through a gaze shift. Neural responses to the cue were assessed with amplitude of the EDAN and ADAN in response to the cue (indicative of attention orienting and holding to the gaze-cued side). In addition, we measured amplitude and latency of the P1 and N200, and behavioral responses including both manual and saccadic reaction times in response to the target. We hypothesized that global visual information has a primary role during gaze-cued orienting of attention: the laterality and gaze cue-validity effect are primarily driven by global visual information and diminished (i.e. attenuated laterality or cue-validity effect) when only local visual information is present.

In contrast to the expectations, there was a gaze cue-validity effect (i.e. valid versus invalid) in all spatial frequency conditions and no interaction effect with spatial frequency content, as measured using amplitude and latency of brain activity, manual reaction times and saccadic reaction times during the localization task. Furthermore, we found no reflections of attention orienting (EDAN), but did find reflections of attention holding (ADAN). However, there was no interaction with spatial frequency content. The lack of interaction effects deviates from results of other studies that investigated reaction times [8,10] and the N200 [6] during a localization task, and contradict the previously suggested primary role of global visual information during gaze-cued orienting of attention. These results are however in line with studies that measured reaction times during a localization task [6], an identification task [9] and a discrimination task [7] and the ADAN [10] and P1 [6,10] during a localization task. These results suggest that gaze-cued orienting of attention is driven by both global and local visual information.

The result, that gaze-cued orienting of attention is possible when either global or local information is available, increases insight in the response of the neural mechanisms related to social gaze. Cortical brain areas involved in social gaze processing are suggested to receive information via two pathways. The magnocellular pathway, tuned to low spatial frequency (global) visual information, projects information via a subcortical fast-track including the amygdala [13,31–33]. The parvocellular pathway, tuned to higher spatial frequency (local) visual information, projects this information via the cortical visual system [13,34]. The present results show a gaze laterality effect and cue-validity effect for both global and local visual information. This suggests that both the magnocellular and parvocellular system are capable of processing information relevant for gaze-cued orienting of attention, resulting in an ADAN and cue-validity effect.

Although our hypothesis that global visual information has a primary role during gaze-cued orienting of attention is rejected, the present results do show that behavioral responses (i.e. manual and saccadic reaction times) towards the target in the HSF and MSF condition are slower compared to the unfiltered condition, whereas responses in the LSF condition do not differ from the unfiltered condition. Previous research has shown that behavioral responses towards higher spatial frequency information (>4cpd; local) are slower than towards lower ones [35,36]. The present results expand this previous research by showing that attention shifting from the gaze cue towards the target is slower when the lower spatial frequency information (<2cpd) is not present in the gaze cue. This result is in line with previous behavioral results that showed slower manual responses towards the target for inverted compared to upright faces [9,10]. Theoretically, the slower response to targets in absence of global information could be due to less or slower attention to the surrounding of the gaze cue stimuli. The option that there is less attention is however rejected by the lack of difference between filtered faces on attention holding (ADAN). Consequently, these results might imply that global visual information speeds up responses towards other entities appearing in the surrounding of gaze cue stimuli. Further research is needed to unravel which neural processes specifically lead to slower responses when the lower spatial frequency information is not present in the gaze cue.

Several factors influencing gaze-cued orienting of attention become apparent when comparing the results between studies (current study; [6–10]). None of these factors individually provide a sufficient explanation of the discrepancies between results. However, they are likely to interactively affect the involvement of local or global information in gaze-cued orienting of attention. Firstly, the measure that is used to investigate gaze-cued orienting of attention might affect the results. We suggested that saccadic and neural responses might be more sensitive and relevant measures, compared to manual responses, to detect differences in gaze-cued orienting of attention. However, we found for manual, saccadic and neural responses that gaze-cued orienting of attention is driven by both local and global visual information. Secondly, age might influence whether social gaze processing is driven by local or global visual information. As sensitivity for spatial frequency information is immature in children [37], this might play a role in the different results between studies. Another factor may be that the stimuli differed between studies: the present study used neutral static stimuli instead of more ecologically valid emotional dynamic stimuli [6]. However, other research reported partly similar results using either static [7,8,10] or dynamic stimuli [6]. Another aspect of the stimuli, being luminance contrast, is suggested to affect processing of information in faces as well [5]. As the four conditions (UF, LSF, MSF, HSF) differ in the amount of luminance contrast present in the faces, effects of contrast on the current findings cannot be excluded. Nevertheless, the present results show that visual information (e.g. the four filter conditions) influences behavioral responses during gaze-cued orienting of attention. Recent studies also differed in the task design (i.e. localization, discrimination or identification) used to measure cue-validity effects, but this did neither lead to a specific pattern in results. Further research should unravel the interacting influence of measure, age, stimuli and task on results regarding the relation between spatial frequency perception and gaze-cued orienting of attention.

There are some limitations to the present study. We did not find neural support for attention orienting (EDAN), although there was attention holding (ADAN). Previous research [21, 22] suggested the use of different experimental parameters could explain the lack of an EDAN in earlier studies [38, 39]. However, in the present study we used the suggested necessary experimental parameters (i.e. face photographs and a target localization task), but found no EDAN. Further research should unravel which factors are necessary to elicit an EDAN. Moreover, the small number of participants and the small number of trials could indicate a lack of power. However, as the effect sizes of the non-significant effects are very small, we believe it is highly unlikely that we would have found different effects with more participants and larger number of trials. Another limitation is the difference in presentation time of the gaze cue between the eye tracking (i.e. 300ms) and the EEG (i.e. 500ms) tasks. Although we find the same results for the behavioral measures (i.e. saccades in the eye-tracking task and manual responses in the EEG task) in both tasks, we cannot be sure the exact same processes are involved. Furthermore, during target presentation the averted-gaze remained visible on the screen to avoid offset effects in the EEG. This task design allowed us to investigate P1 and N200 in response to the target. Probably the facial P1 and N200, and any effects of spatial frequency hereupon, are already faded out, and are unlikely to have a direct effect on the P1 and N200 in response to the target. However, because the filtered face is still present on the screen during target presentation, we cannot rule out that this influences the P1 and N200 in response to the target. This could have complicated interpretation of possible filtering effects. New research could consider adding a gap between the presentation of the face and presentation of the target, with the risk of inhibition of return effects and the downside of creating a slower, and thereby possibly less ecological valid, design. Lastly, whereas the present study yielded not enough power to investigate overt shifts of attention in response to the gaze cue (i.e. anticipatory saccades), this might be an interesting direction for further research.

To conclude, the present study shows with multiple measures and well-described stimuli that in adults global visual information does not have a primary role during gaze-cued orienting of attention, which replicates and extends previous research (e.g. [7,9]). The present study does show, similar to previous research (e.g. [9,10]), that in adults the speed of behavioral responses towards peripheral targets is lower when most of the global visual information (i.e. LSF) is not present in the gaze cue. As such, global visual information probably is still important for quality of social interaction.

## Supporting Information

S1 Dataset Dataset used for statistical analyses. For each participant gender, age and EDAN, ADAN, P1 and N200 amplitude (VEEG), P1 and N200 latency (LEEG), median saccadic reaction times (ET) and median manual reaction times (EEGRT)—towards invalid (I) and valid (V) targets that were preceded by faces that were either unfiltered (U) or filtered to contain the lower spatial frequencies (L), mid spatial frequencies (M) of high spatial frequencies (H)—were reported.
(XLSX)

S1 Fig Cue-validity * filter. Mean ± SE of P1 amplitude, P1 latency, N200 amplitude, N200 latency, manual reaction times (RT) and saccadic reaction times (RT) for each cue-validity (i.e. invalid (I) and valid (V)) and filter condition (i.e. unfiltered (UF) or filtered to contain the lower spatial frequencies (LSF), mid spatial frequencies (MSF) of high spatial frequencies (HSF)).
(TIF)

S2 Fig Laterality * filter. Mean ± SE of EDAN and ADAN amplitude for each hemisphere, laterality (i.e. contralateral (C) and ipsilateral (I)) and filter condition (i.e. unfiltered (UF) or filtered to contain the lower spatial frequencies (LSF), mid spatial frequencies (MSF) of high spatial frequencies (HSF)).
(TIF)
