# Supplementary figures and images for: The Role of Global and Local Visual Information during Gaze-Cued Orienting of Attention
Source: PLoS One. 2016 Aug 25;11(8):e0160405. doi: 10.1371/journal.pone.0160405 (PMC4999176; doi:10.1371/journal.pone.0160405)

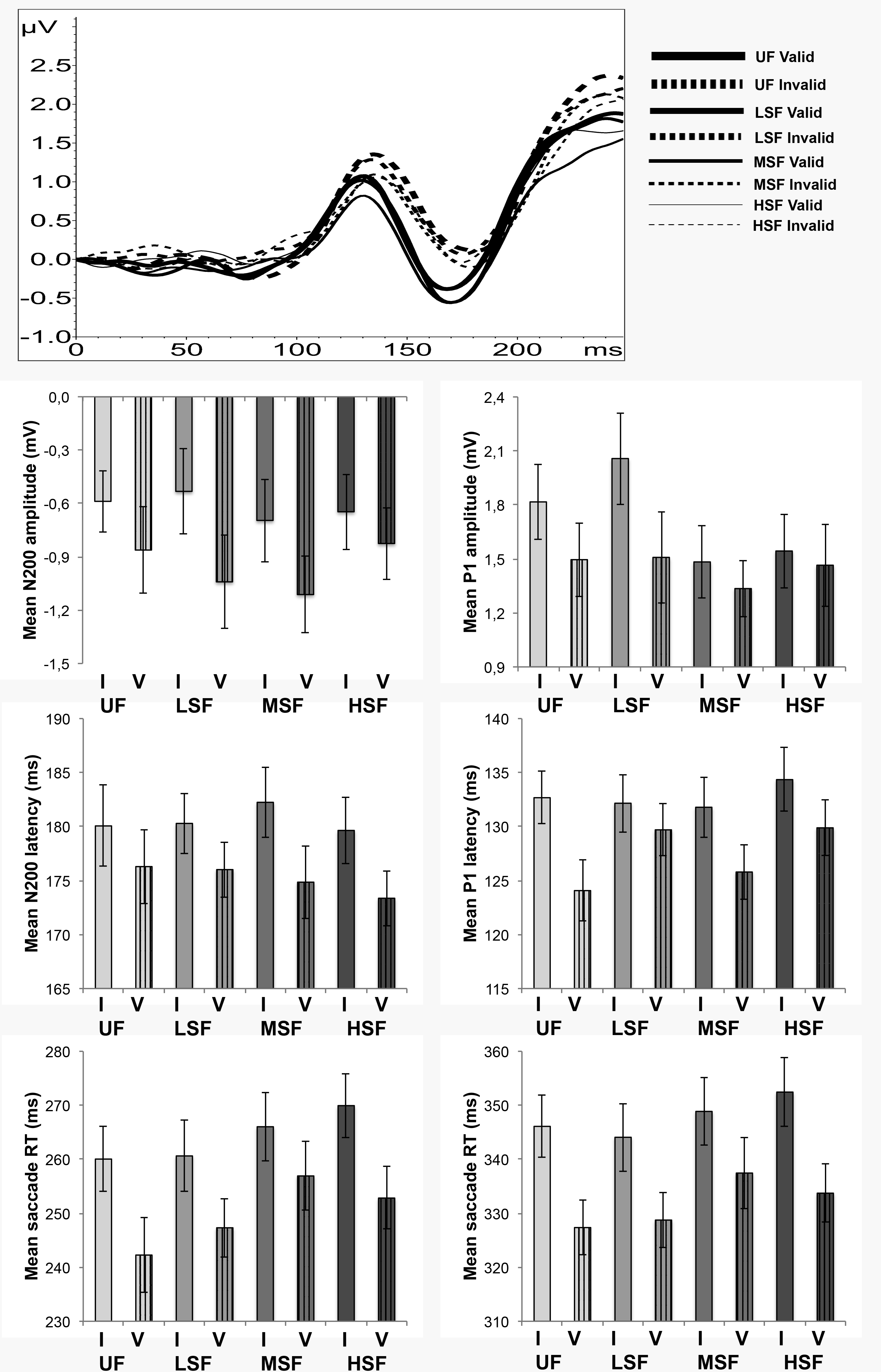

Supplement: S1 Fig — Mean ± SE of P1 amplitude, P1 latency, N200 amplitude, N200 latency, manual reaction times (RT) and saccadic reaction times (RT) for each cue-validity (i.e. invalid (I) and valid (V)) and filter condition (i.e. unfiltered (UF) or filtered to contain the lower spatial frequencies (LSF), mid spatial frequencies (MSF) of high spatial frequencies (HSF)). (TIF) [file pone.0160405.s002.tif]

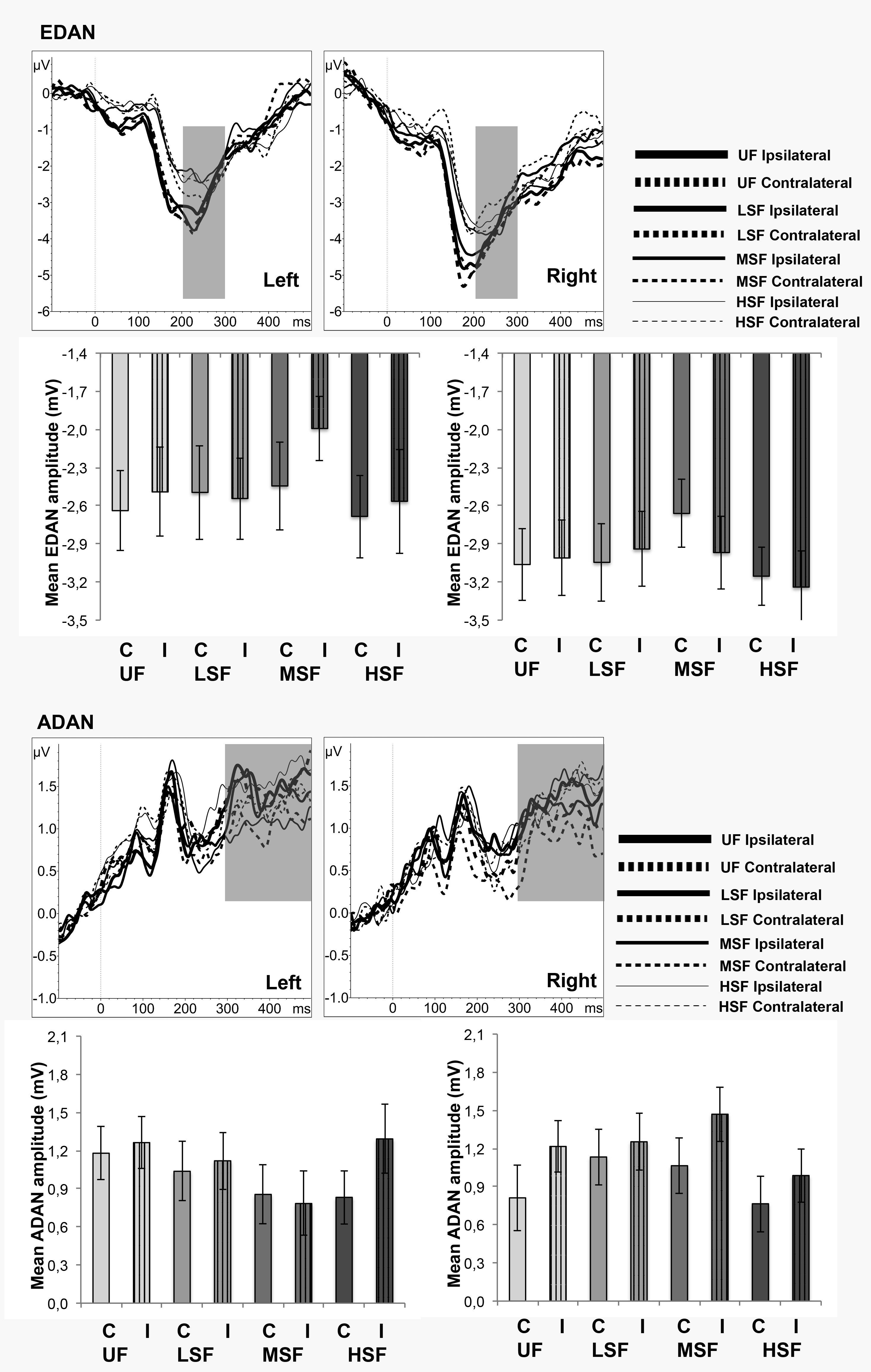

Supplement: S2 Fig — Mean ± SE of EDAN and ADAN amplitude for each hemisphere, laterality (i.e. contralateral (C) and ipsilateral (I)) and filter condition (i.e. unfiltered (UF) or filtered to contain the lower spatial frequencies (LSF), mid spatial frequencies (MSF) of high spatial frequencies (HSF)). (TIF) [file pone.0160405.s003.tif]
